# Comparison of the efficacy of pulsed radiofrequency in treating acute herpetic neuralgia and postherpetic neuralgia in the thoracic segment

**DOI:** 10.3389/fneur.2024.1425796

**Published:** 2024-08-29

**Authors:** Huan Wang, Dandan Zhang, Shiyu Wang, Hui Wang, Huiyong Nie

**Affiliations:** ^1^Department of Pain Management, The First Affiliated Hospital of Xi'an Jiaotong University, Xi'An, China; ^2^Department of Geriatric Cardiology, The First Affiliated Hospital of Xi'an Jiaotong University, Xi'An, China

**Keywords:** pulsed radiofrequency, herpes zoster, postherpetic neuralgia, dorsal root ganglia, neuropathic pain, sleep quality

## Abstract

**Objectives:**

This study aimed to compare the efficacy of pulsed radiofrequency (PRF) to dorsal root ganglia (DRG) in treating acute herpetic neuralgia (AHN) and postherpetic neuralgia (PHN) in the thoracic segment.

**Methods:**

A total of 243 patients with thoracic herpes zoster-related pain (AHN or PHN) from January 2020 to September 2022 were retrospectively analyzed. They were divided into two groups based on the timing of PRF after herpes zoster onset: an acute herpetic neuralgia group (within 90 days) and a postherpetic neuralgia group (more than 90 days). All patients were treated with PRF at the thoracic DRG. The Visual Analog Scale (VAS), the Athens Insomnia Scale (AIS), the Generalized Anxiety Disorder-7 items (GAD-7), and the Patient Health Questionnaire-9 items (PHQ-9) scores were assessed before and at 1 week, 1 month, 3 months, 6 months, and 12 months after surgery, and the results were then compared between the two groups.

**Results:**

Postoperative scores of VAS, AIS, GAD-7, and PHQ-9 in both groups were significantly lower than preoperative scores (*P* < 0.001). From 1 month to 12 months after surgery, the AHN group showed significantly lower VAS, AIS, GAD-7, and PHQ-9 scores compared to the PHN group (*P* < 0.001). In the AHN group, there was a gradual improvement in these scores from 1 week to 12 months post-surgery. Conversely, the PHN group's scores began to worsen slowly from 1 week to 12 months post-surgery. Over time, the difference in scores between the two groups also increased gradually.

**Conclusion:**

PRF to the DRG is an effective treatment for patients with AHN or PHN who do not respond well to conventional treatments. For AHN patients, PRF to the DRG significantly enhances early pain control, improves sleep and psychological status, and may even prevent the development of PHN.

## Introduction

Herpes zoster (HZ) is an acute skin disease caused by the reactivation and replication of the varicella-zoster virus (VZV) in the dorsal root ganglion (DRG) (1). It mainly manifests as clustered blisters distributed along nerve segments on one side of the body, accompanied by pain and paresthesia during the acute/subacute phase, which is the first 3 months after the onset of shingles (2). The pain during this period is called acute herpetic neuralgia (AHN).

Postherpetic neuralgia (PHN) is the final stage (more than 3 months) of VZV infection and is characterized by severe refractory neuropathic pain (3). Preventing the transition from AHN to PHN is a fundamental therapeutic principle, especially for older patients at an early stage (< 3 months) (2, 4, 5). Both pulsed radiofrequency (PRF) and short-term spinal cord stimulation (stSCS) have proven to be effective in relieving AHN (6). The DRG contains many receptor channels and is an important hub for many nociceptive signal transduction. The proximal end of the DRG nerve cell body extends to the dorsal horn of the spinal cord (7, 8). Persistent abnormal electrical activity in the spinal cord through the DRG can lead to neuropathic processes, such as central sensitization, and increase the risk of PHN development (9). Therefore, the DRG is a crucial target for treating herpes zoster-related pain.

PRF applied to the DRG has been shown to be an effective treatment in studies on herpes zoster-associated pain (AHN or PHN), especially for intractable pain in the thoracic segment. The application of PRF to the DRG is recommended for pain control and prevention of PHN (9). The effect of PRF on the DRG in PHN patients has been reported (10). PRF to the DRG is more effective in the treatment of acute herpetic pain than PHN. Early intervention of PRF in the treatment of AHN may be more helpful in controlling pain and preventing PHN. Although studies have explored PRF therapy for acute herpes zoster pain, large sample sizes and long-term follow-up studies are lacking. This study retrospectively analyzed the clinical effect of PRF on the DRG in treating AHN and PHN.

## Materials and methods

### Patients

**Figure 1:**
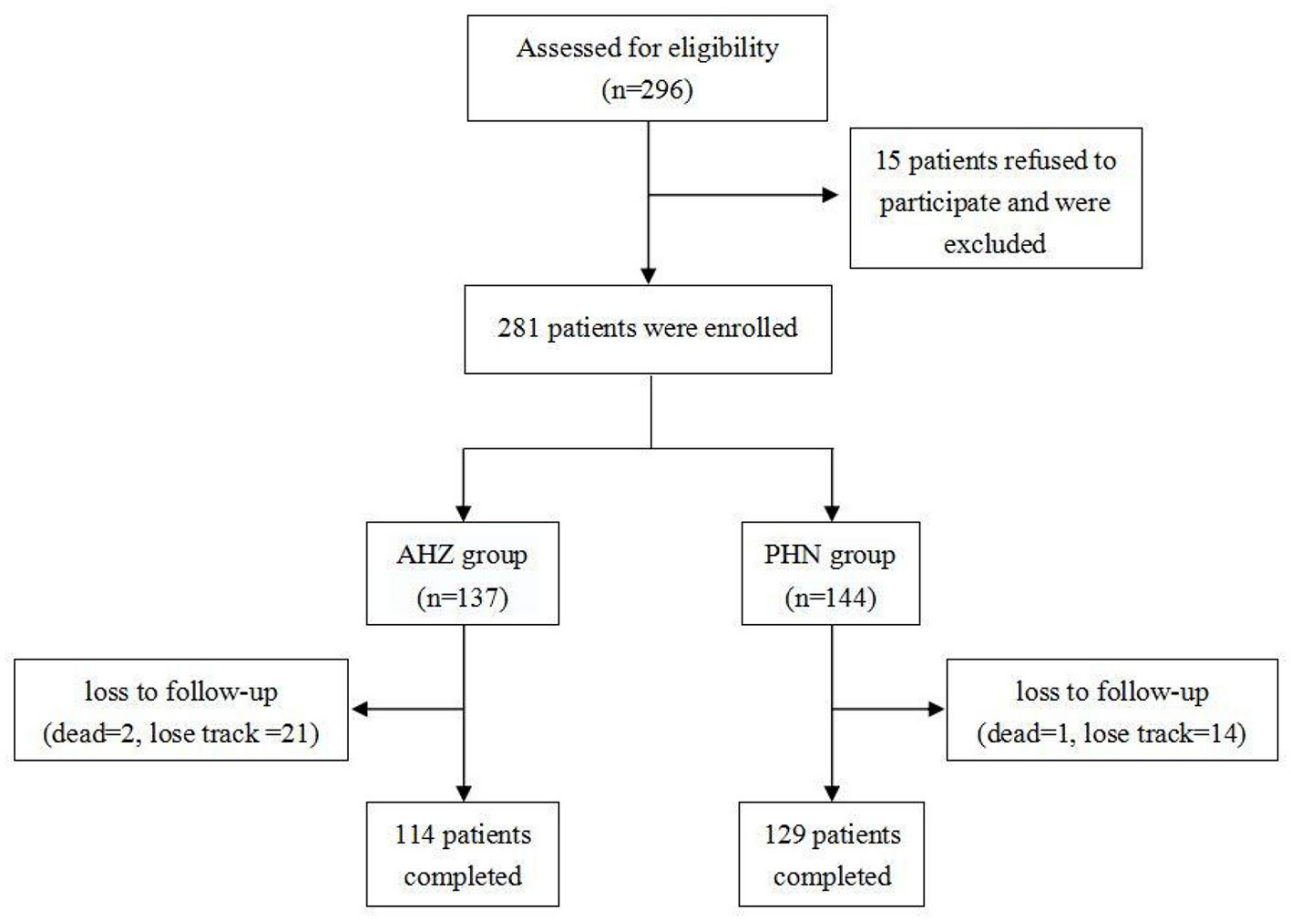
Flow chart of case enrollment and follow-up.

Medical records were obtained from all patients who received pulsed radiofrequency therapy for herpes-related pain (AHN or PHN) from January 2020 to September 2022. We included the medical records of 243 patients with herpes zoster-related pain (AHN or PHN) in a thoracic segment (Figure 1). A total of 281 patients were included before follow-up, of which 38 were lost to follow-up (three deceased and 35 lost contact). Data such as sex, age, height, weight, BMI, preoperative VAS, smoking status, drinking status, hypertension, diabetes, insomnia, and postoperative oral medication of patients were recorded.

Pain intensity (measured using the Visual Analog Scale, VAS), sleep quality (measured using the Athens Insomnia Scale, AIS), and psychological burden (measured using the Generalized Anxiety Disorder-7 items, GAD-7, and the Patient Health Questionnaire-9 items, PHQ-9) of patients were followed up at 1 week, 1 month, 3 months, 6 months, and 12 months after the operation. The therapeutic effects between the two groups were compared.

The study was approved by the Ethics Committee of the First Affiliated Hospital of Xi'an Jiaotong University (KYLLSL-2022-205).

Patients were included if they met the following inclusion criteria: (1) Met the diagnostic criteria of HZ and PHN; (2) the lesion occurred in the thoracic spinal nerves; (3) persistent intense pain, with/without local skin hyperalgesia, sensory abnormalities; (4) pain levels not well controlled by standard pharmacotherapy (antiepileptic drugs, antidepressants, lidocaine patches, opioids, etc.); and (5) 24-h VAS ≥5. After the preliminary selection, patients were excluded if they had the following exclusion criteria: (1) unwillingness or inability to complete the follow-up; (2) severe coagulation disorder; (3) severe liver or kidney dysfunction; (4) history of drug abuse; (5) severe cardiopulmonary disease; or (6) intellectual inability to complete self-evaluation using VAS or AIS.

### Surgical procedure

**Figure 2:**
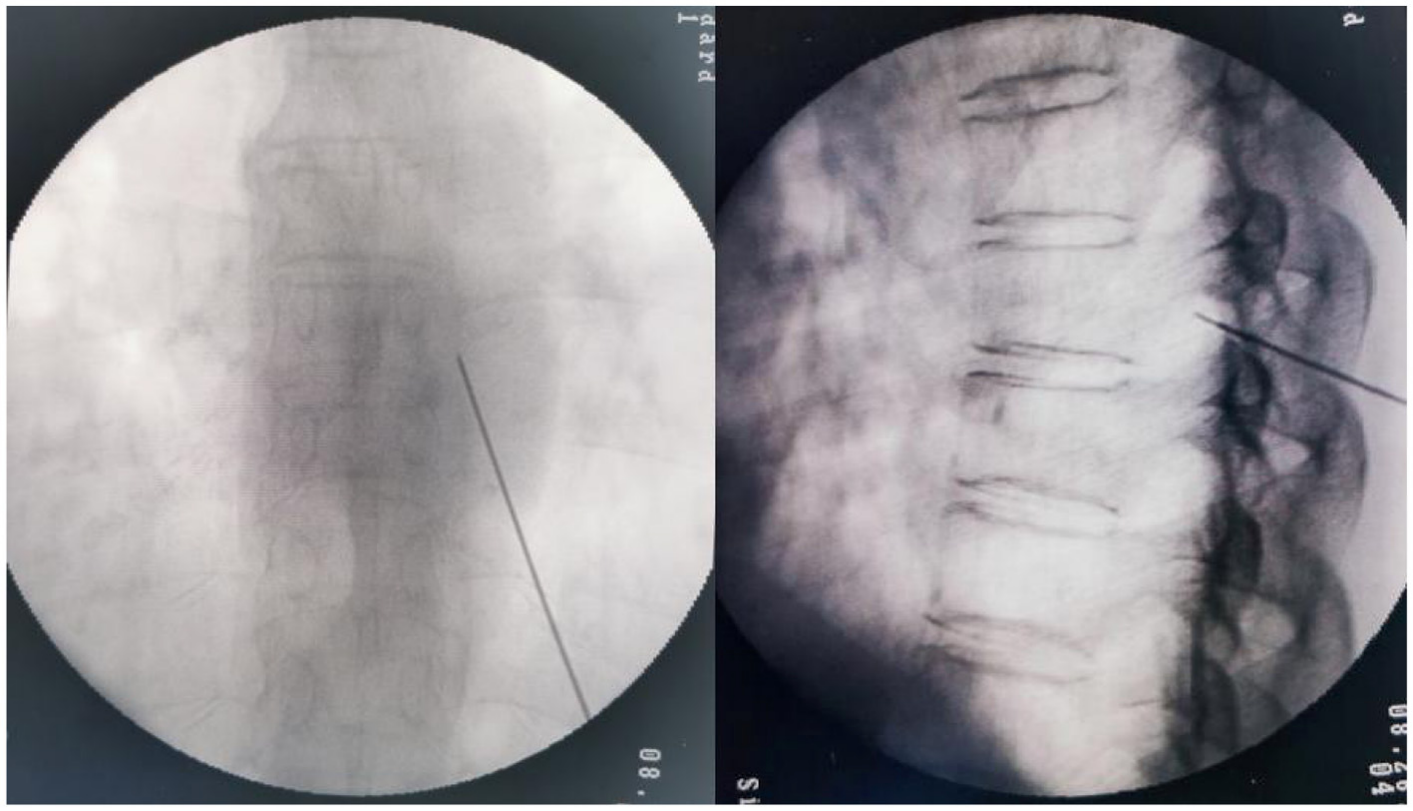
Puncture of the thoracic DRG under the guidance of an X-ray. The left is the anteroposterior view, and the right is the lateral view.

The application of PRF to the DRG in treating all patients with either AHN or PHN in the thoracic segment was performed by the same team of doctors. Continuous ECG monitoring was carried out throughout the procedure, and the diseased spinal nerve was preoperatively determined, based on the patient's pain site. The patient was in the prone position for the surgical procedure. The surgical area was disinfected routinely. Under local infiltration anesthesia, a needle with a 5 mm exposed tip was inserted layer by layer into the DRG of the spinal nerve under C-arm guidance (Figure 2). Upon reaching the expected position, a high-frequency (50 Hz, 500 μs width, < 0.2 volts) sensory stimulation test and a low-frequency (2 Hz, 1,000 μs width, < 0.5 volts) motor stimulation test were conducted. These tests confirmed the accurate placement of the needle tip. Subsequently, the treatment was carried out with PRF parameters set at 42°C, 2 Hz, with a field intensity of 90 V, for a duration of 900 s.

During follow-up, it was found that some patients continued to take oral medications, such as pregabalin and opioids, due to poor response to PRF. These cases were recorded and analyzed. However, due to the extended follow-up period, accurate information on the oral doses and treatment courses of the drugs for many patients could not be obtained. Therefore, the number of patients who continued to take medications was used as one of the indicators for comparing the efficacy between the groups. Even though this method introduces some systematic bias, it still provides valuable insights into the difference in treatment effect between the two groups.

### Efficacy evaluation and follow-up

The Visual Analog Scale (VAS) was used to evaluate the level of pain, with 0 indicating no pain and 10 indicating maximum pain. The patient selected a number between 0 and 10 on a caliper to indicate the intensity of their pain.

The Athens Insomnia Scale (AIS) (11) was used to assess sleep quality. An AIS score of 6 was considered insomnia, scores between 4 and 6 suggested suspected insomnia, and scores below 4 indicated no sleep disturbance.

The Generalized Anxiety Disorder-7 item (GAD-7) (12) scale and the Patient Health Questionnaire-9 items (PHQ-9) (13) were used to evaluate anxiety and depression, respectively. The scores ranging from 0 to 4 indicated normal levels, while the scores ranging from 5 to 9 indicated symptomatic levels, with higher scores indicating more severe symptoms, up to a maximum score of 27.

Follow-up mainly consisted of outpatient visits, supplemented by telephone and online follow-ups. Patients were asked to fill out the evaluation forms honestly. Follow-up sessions were scheduled before operation, 1 week, 1 month, 3 months, 6 months, and 12 months post-operation.

### Statistical analysis

SPSS 23.0 statistical software was used for analysis. Data were expressed as mean ± standard deviation. A *t*-test was performed to measure the data such as age, height, etc. Scores from the VAS, AIS, GAD-7, and PHQ-9 were compared between the two groups using repeated-measures analysis of variance. The chi-squared test was performed to compare categorical data such as sex, hypertension, diabetes, and oral medications. A *P-*value of < 0.05 was considered statistically significant.

## Results

### General information of the AHN group and the PHN group

Twelve months after surgery, the number of patients requiring oral medication (pregabalin or opioids) in the PHN group was 3.3 times greater than in the AHN group, indicating a statistical difference (*P* = 0.001). There were no statistically significant differences in terms of patients' basic information, including sex, age, height, weight, BMI, VAS, smoking, drinking, hypertension, diabetes, and insomnia, between AHN group and PHN group (see Table 1).

**Table 1:** General information of the AHN group and the PHN group.

	**AHN group (*n* = 114)**	**PHN group (*n* = 129)**	***P*-value**
Sex (M/F)	46/68	59/70	0.398
Age (year)	70.4 ± 7.6	71.4 ± 7.5	0.313
**Height (cm)**			
Male	170 ± 6	169 ± 7	0.591
Female	158 ± 5	158 ± 7	0.895
**Weight (kg)**			
Male	70 ± 6	70 ± 7	0.326
Female	62 ± 5	61 ± 5	0.216
BMI	23.0 ± 3.0	23.1 ± 3.3	0.883
VAS	6.8 ± 0.8	6.8 ± 0.7	0.924
Smoking (*n*, %)	31 (27.2)	38 (29.5)	0.696
Drinking (*n*, %)	24 (21.1)	28 (21.7)	0.901
Diabetes (*n*, %)	15 (13.1)	21 (16.3)	0.494
Hypertensive (*n*, %)	23 (20.1)	32 (24.8)	0.389
Insomnia (*n*, %)	35 (30.7)	47 (36.4)	0.098
Oral medications (*n*, %)	10 (8.8)	33 (25.6)	0.001
Pregabalin	6	21	
Opioids	4	12	

### Postoperative VAS changes in the AHN group and the PHN group

**Figure 3:**
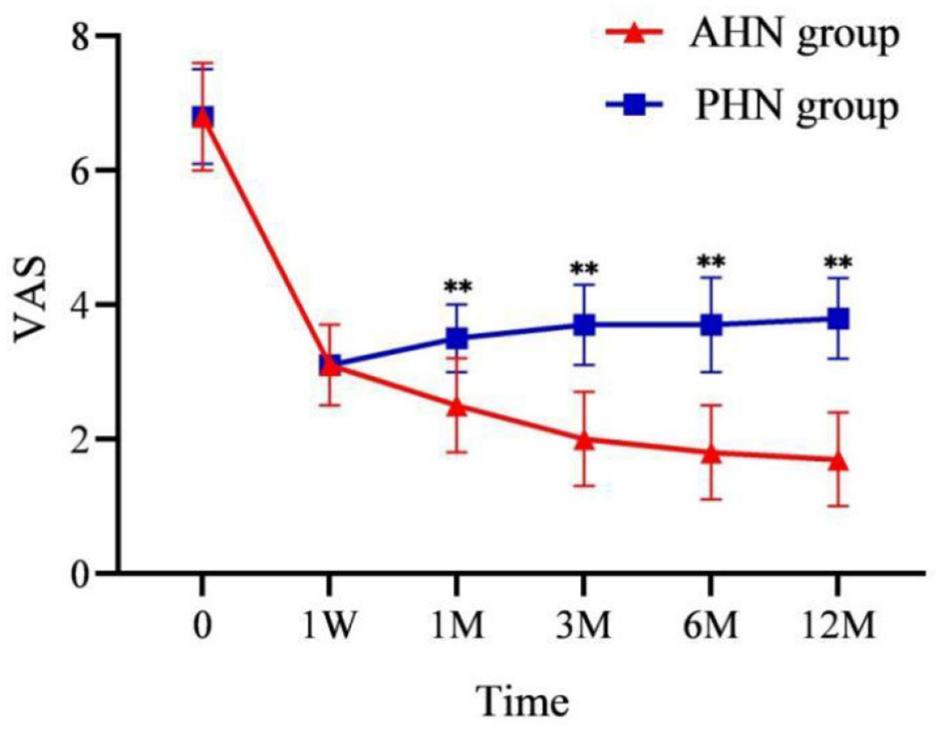
Changes of VAS in the AHN group and the PHN group. **P < 0.001. AHN group, *n* = 114; PHN group, *n* = 129. VAS, Visual Analog Scale. Time: postoperative follow-up time. W, week; M, month. 0 = preoperative.

The VAS scores of both the AHN and PHN groups were significantly lower than those before the procedure (*P* < 0.001). In addition, the VAS score of the AHN group gradually decreased over time, while the PHN group's VAS score gradually increased from 1 week to 12 months after surgery (*P* < 0.001). The difference between the two groups gradually increased after 1 month (Figure 3). The results of repeated-measures analysis of variance showed a statistically significant time effect between the two groups (*F* = 1,872.990, *P* < 0.001), indicating that VAS scores in both groups significantly reduced over time compared with those before surgery. Time^*^group effect was also statistically significant (*F* = 164.289, *P* < 0.001), indicating that the decrease in VAS scores in the AHN group was significantly greater than that in the PHN group.

### Postoperative changes in AIS, GAD-7, and PHQ-9 scores in the AHN and PHN groups

**Figure 4:**
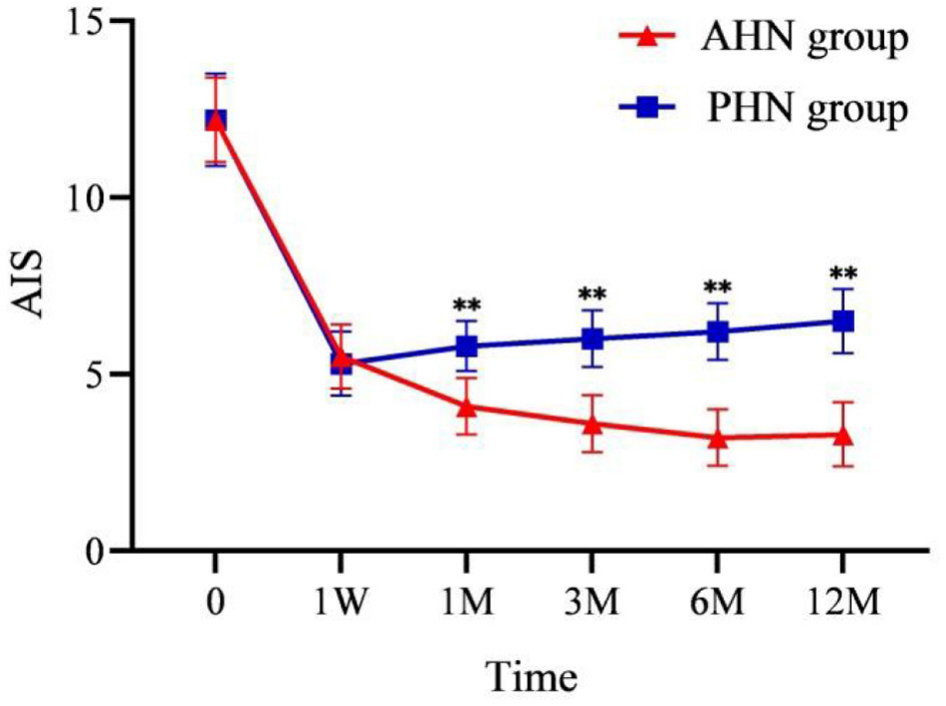
Changes of AIS in the AHN group and the PHN group. **P < 0.001. AHN group, *n* = 114; PHN group, *n* = 129. AIS, Athens Insomnia Scale. Time: postoperative follow-up time. W, week; M, month. 0 = preoperative.

**Figure 5:**
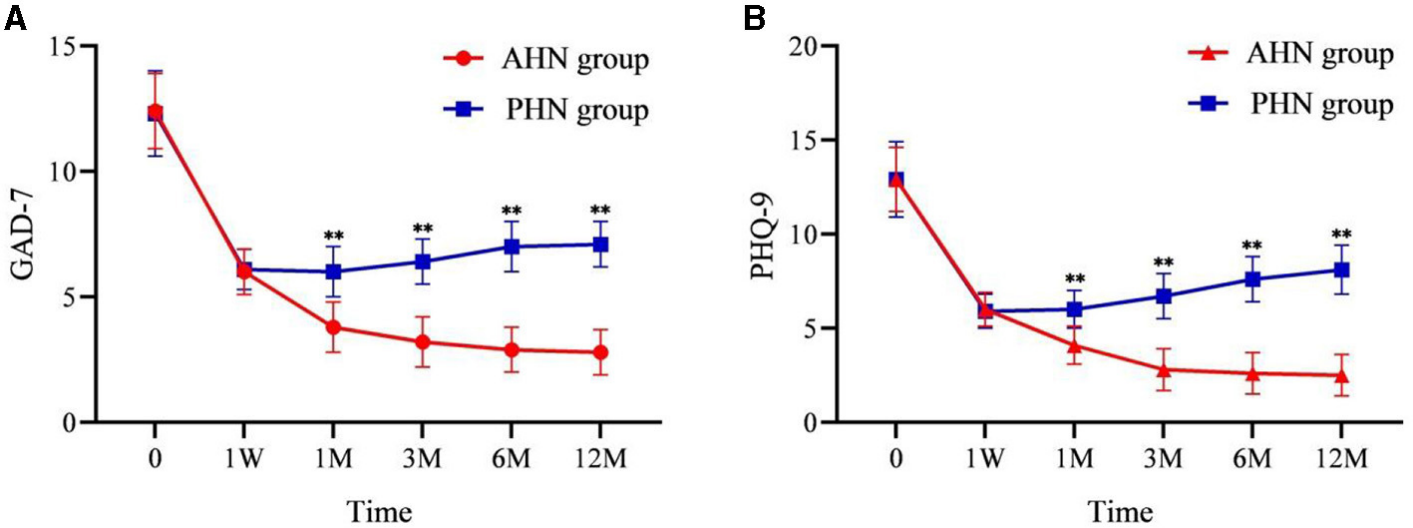
Changes of GAD-7 **(A)** and PHQ-9 **(B)** in the AHN group and the PHN group. **P < 0.001. AHN group, *n* = 114; PHN group, *n* = 129. GAD-7, Generalized Anxiety Disorder-7 items; PHQ-9, Patient Health Questionnaire-9 items. Time: postoperative follow-up time. W, week; M, month. 0 = preoperative.

AIS, GAD-7, and PHQ-9 scores significantly decreased after surgery compared to preoperative scores (*P* < 0.001). In the AHN group, AIS, GAD-7, and PHQ-9 scores decreased over time following the procedure. In contrast, the PHN group's AIS, GAD-7, and PHQ-9 scores gradually increased at all time points post-procedure. From 1 to 12 months after surgery, the differences between the two groups became significant (*P* < 0.001) and increased over time (Figures 4, 5A, B).

Repeated-measures analysis of variance of AIS, GAD-7, and PHQ-9 scores yielded similar results to those of VAS. The time effect has a statistical difference (*F* = 3,413.527, *P* < 0.001; *F* = 2,366.545, *P* < 0.001; *F* = 2,384.951, *P* < 0.001), and the time^*^group interaction effect was also significant (*F* = 228.316, *P* < 0.001; *F* = 266.207, *P* < 0.001; *F* = 392.077, *P* < 0.001). This indicates that there were significant differences in AIS, GAD-7, and PHQ-9 scores between the two groups before and after surgery. Moreover, there were significant differences between the groups (*P* < 0.001), with the AHN group showing a more substantial decrease in AIS, GAD-7, and PHQ-9 scores compared to the PHN group.

## Discussion

PHN is a chronic neuropathic pain that is an unpredictable sequela of herpes zoster, which is induced by the varicella-zoster virus. It is usually seen in the elderly and the immunocompromised individuals (2, 14). PHN is defined as pain that lasts for more than 3 months after the recovery of herpes zoster, which is characterized by local skin allodynia or hyperalgesia (15). The pain is typically described as burning and tingling sensation and can be accompanied by numbness, leading to psychosocial dysfunction and a negative impact on the quality of life (5). Prevention of PHN can be achieved by vaccinating high-risk groups against herpes zoster, the early and regular use of antiviral drugs, and the aggressive and effective treatment of pain in the early stages of acute herpes zoster (16–19).

Studies have reported that early and effective pain intervention treatment may reduce pain and complications associated with herpes zoster and PHN (20, 21). Once PHN occurs, the pain usually persists for months to years, with the longest observed case being 20 years. Although the current first-line drugs for the treatment of PHN (4, 22–25), such as pregabalin, gabapentin, and a 5% lidocaine patch, can relieve pain, some patients do not experience adequate relief. Opioids have also long been prescribed to patients with PHN to reduce pain (26). Therefore, in this study, patients with severe pain after PRF surgery still required oral pregabalin or opioids, and the number of PHN patients requiring oral drugs was higher than that of AHN patients.

It should be noted that due to the long follow-up period, many patients could not accurately recall the dosage and duration of pregabalin and opioids. However, they could remember whether they took these drugs. This affects the integrity of the data, but valuable insights can still be obtained by comparing the number of patients taking oral medications in the two groups. Future studies should record oral medication dosages more meticulously.

PRF therapy is an effective complementary method for pain relief, being a grade B recommended therapy for the DRG (10, 27). Clinically, PRF can relieve AHN and PHN to a certain extent. Studies have suggested that early pain management of herpes zoster can reduce pain and prevent PHN (9, 28, 29). Further analysis of the therapeutic effects of PRF to the DRG in patients with AHN or PHN is beneficial in assisting clinicians to select a more suitable timing for pain treatment.

Histopathological studies have found that patients with severe PHN experience the loss of cells, axons, and myelin in the sensory ganglia, accompanied by fibrosis (30). This suggests that pain perception in patients with PHN may be caused by the ectopic firing of nociceptors and low-threshold afferents in the dorsal root ganglion (31). PRF targeting the DRG can more effectively relieve pain caused by varicella-zoster virus invading the skin.

In our study, we followed up with 114 patients with AHN and 129 patients with PHN. To control pain and prevent PHN as much as possible, the researchers focused on treating pain in the acute phase of HZ (within 3 months after the onset of herpes zoster). The results showed that PRF to the DRG resulted in significantly more effective pain relief for AHN compared to PHN.

In the study of PRF in treating PHN, Han et al. (32) suggested that high-voltage PRF could offer better analgesic effects. PRF can be effectively used for treating PHN in the upper limbs because it operates at a safe temperature of 42°C, providing a protective effect on the nerve root. In addition, high-voltage, long-duration PRF to the DRG is effective and safe for treating HZ neuralgia in the subacute stage (33).

Aggarwal et al. (28) showed that early pain control can significantly improve sleep quality in patients with HZ. This finding is similar to our results. In our study, postoperative pain relief was more significant in the AHN group than in the PHN group, resulting in a considerable improvement in sleep quality for AHN patients. Conversely, the sleep quality of PHN patients did not improve as significantly as that of AHN patients. This disparity may be due to the long-term pain experienced by PHN patients, which severely affects sleep quality (34). Yamada et al. (35) pointed out that insufficient sleep may be a new risk factor for PHN.

Chronic pain imposes a severe psychological burden on patients (36–39). In our study, anxiety (measured using the GAD-7 scale) and depression (measured using the PHQ-9) in patients within the AHN group improved with pain relief and were significantly better than those in the PHN group. This indicates that the effective control of pain in acute herpes zoster can better reduce the psychological burden on patients. The study emphasizes the importance of early diagnosis and treatment of zoster-related pain (40).

Although rare, complications of PRF therapy to the DRG can be potentially severe. The risk of paraplegia due to injury to the Adamkiewicz artery during PRF therapy to the DRG cannot be ruled out (41). Similarly, ultrasound is of great value in detecting blood vessels and monitoring motor-evoked potentials during PRF-DRG. It can detect early signs of spinal cord ischemia, allowing the procedure to be stopped in time (42).

### Limitations of the study

Our study has some limitations. As a retrospective study, it may introduce some bias in the data and lack the rigor of multicenter randomized controlled studies. Currently, there is no international standard for setting the parameters of pulsed radiofrequency therapy. Conducting a multicenter, prospective, double-blind controlled study is the next step worth pursuing.

## Conclusion

PRF to the DRG is an effective treatment for patients with AHN or PHN who do not respond well to conventional treatments. For AHN patients, PRF to the DRG significantly enhances early pain control, improves sleep and psychological status, and may even prevent the development of PHN.

## Data Availability

The original contributions presented in the study are included in the article/supplementary material, further inquiries can be directed to the corresponding author.
